# COVID-19: A Matter of Planetary, not Only National Health

**DOI:** 10.4269/ajtmh.20-0419

**Published:** 2020-05-18

**Authors:** Ashley Jowell, Michele Barry

**Affiliations:** 1 Stanford University School of Medicine, Stanford, California;; 2 Center for Innovation in Global Health, Stanford University, Stanford, California

## Abstract

The COVID-19 pandemic highlights the multidimensional and inseparable connection between human health and environmental systems. COVID-19, similar to other emerging zoonotic diseases, has had a devastating impact on our planet. In this perspective, we argue that as humans continue to globalize and encroach on our surrounding natural systems, societies must adopt a “planetary health lens” to prepare and adapt to these emerging infectious diseases. This piece further explores other critical components of a planetary health approach to societal response, such as the seasonality of disease patterns, the impact of climate change on infectious disease, and the built environment, which can increase population vulnerabilities to pandemics. To address planetary health threats that cross international borders, such as COVID-19, societies must practice interdependence sovereignty and direct resources to organizations that facilitate shared global governance, and thus can enable us to adapt and ultimately build a more resilient world.

Our planet faces a rapidly evolving pandemic that is transforming life as we know it. As the number of COVID-19 cases and mortalities skyrocket across the globe, governments and health systems are scrambling to stem the pandemic. Amid all of this, a question looms: What will happen to COVID-19 patterns as the seasons change and transition to summer in the Northern Hemisphere and winter in the Southern Hemisphere? This question highlights the complex, interconnected relationship between humans and our environment. To prepare and adapt to emerging infectious diseases such as COVID-19, we must adopt a “planetary health lens” that recognizes the multidimensional and inseparable connection between human health and natural systems which goes beyond national issues.^1^

The origin story of COVID-19 highlights the proximity between human well-being and our surrounding environment. COVID-19 is a zoonotic disease originating in bats. Although the exact transmission pathway between amplifier animals and humans is still being studied, scientists have traced the first human cases of COVID-19 to the Huanan Seafood Wholesale Market in Wuhan, China, in December 2019. Here, the global pandemic presumably began via an intermediate animal host that first transmitted the virus to humans or via direct human contact from an ill vendor at the market who had traveled from a local farm with animals and bats comingling.

COVID-19 is not the first example of an emerging zoonotic infection that has had devastating impacts on global human health. In recent decades, humans have altered surrounding habitats and increased deforestation to make room for growing populations, extract natural resources, and build farms. This altered ecology puts societies in closer proximity to potential hosts of diseases such as bats, which augments the risk of emerging zoonotic diseases. Both the 2002–2003 severe acute respiratory syndrome (SARS) coronavirus and 2014–2016 Ebola virus epidemics were zoonotic diseases that transmitted into human hosts from bats.^2^ Altered land use engendering close human/animal interfacing, coupled with globalization and increased population density, heightens the risk that emerging zoonotic diseases transform into pandemics.

The 1998 Nipah virus outbreak is a powerful example of how altered land use can cause a lethal outbreak. In Malaysia, deforestation, plantations, and drought resulted in fruit bats migrating to cultivated fruit orchards containing piggeries. This close existence between fruit bats, later identified as the reservoir host of Nipah virus, and pigs enabled Nipah virus transmission between these animals. The pigs then served as an intermediate host that rapidly spread the virus throughout pig farms across Malaysia. Pigs consequently transmitted the Nipah virus through secretions to humans, which manifested as an encephalitis, with a nearly 40% mortality rate.^3^ As humans continue to globalize and encroach on surrounding environments, communities will be at risk of viral spillover and a mounting number of emerging zoonotic diseases.

Cyclic seasonal patterns play an enormous role in the transmission of pathogens, and the effect that this will have on the COVID-19 pandemic is currently unknown. Pathogens survive and are transmitted under diverse environmental conditions, and therefore peak at different times of the year in different geographical locations. For example, the *Aedes aegypti* mosquito that transmits dengue, chikungunya, and Zika thrives and is a more aggressive biter in warm temperatures and high humidity. As the world’s climate changes, this mosquito’s habitat will shift poleward and into higher altitude regions of the tropics, whereas some environments where these vectors are endemic might become too warm to remain habitable.^4^

Seasonality also plays an important role in respiratory viral transmission, leading to infection peaks at different times of the year (Figure 1). Consider the influenza virus, which peaks during the winter in temperate climates because of a number of factors. For example, the literature suggests that low wintertime humidity results in greater influenza viral stability in airborne respiratory droplets, which could augment aerosol transmission.^5^ Cold temperatures and lack of sunlight also dampen the host immune response through impeded mucociliary clearance, damaged airway epithelial cells, and potential diminished vitamin D immune modulation.^6,7^ Finally, winter months often correlate with indoor crowding and holiday travel, in turn increasing the risk of viral transmission. By contrast, in warm and humid tropical climates, influenza has been hypothesized to be primarily transmitted less by aerosolized droplets and more via direct contact with secretions. This hypothesis is consistent with the more sporadic pattern of influenza outbreaks seen throughout the year in the tropics.^6,8^

**Figure 1.**
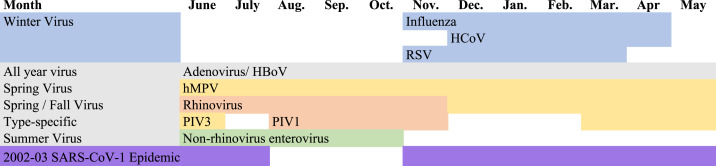
Adapted from Moriyama et al.^6^ Seasonality of respiratory virus infections in temperate regions. HBoV = human bocavirus; HCoV = human coronavirus; hMPV = human metapneumovirus; PIV = parainfluenza virus; RSV = human respiratory syncytial virus.

Scientists are working to determine how seasonality might impact the transmission of COVID-19, which is challenging, as it is a novel virus with no prior human immunity. The last six influenza pandemics have all begun in the spring or summer of the Northern Hemisphere, indicating that pandemic spread might not follow traditional seasonal patterns.^6^ No one understands the seasonality of SARS-CoV-1 and why it disappeared at the end of the summer in 2003, never to reappear as of yet. There are a handful of unpublished reports modeling potential links among temperature, humidity, and COVID-19. However, there is currently no consensus on the relationship between these factors, particularly as COVID-19 is spreading in countries with a range of temperatures and humidities. The climate crisis could further complicate the spread of COVID-19. If a natural disaster, such as a wildfire or hurricane, requires evacuation from hospitals or homes, how might this impact COVID-19 treatment efforts or physical distancing to reduce transmission? Models are unable to take into account region-specific, socio-environmental factors and public health responses; therefore, the question of how transmission will change with the seasons remains.

Crowded, dense, and polluted built environments, which are often inhabited by poor and marginalized communities, can increase population vulnerabilities to epidemics such as COVID-19. Increased exposure to air pollution and smoking have been associated in these impoverished communities, leading to high risks of noncommunicable diseases (NCDs) such as coronary artery disease (CAD) and chronic obstructive pulmonary disease (COPD). The literature suggests that COPD, smoking, CAD, heart failure, and cardiac arrythmia in hospitalized COVID-19 patients are associated with a higher risk of in-hospital death.^9^ The precise impact of other NCDs on COVID-19 progression remains a question. In addition, populations in these environments are less able to shelter from COVID-19, seek care when infected, or access water for handwashing to prevent disease transmission. The rapid spread of COVID-19 is a firsthand example of how threats such as pandemics, climate change, and air pollution ignore country borders and can spread across the globe. It is critical that countries unite together, develop cohesive responses, and build capacity to control such threats—a concept that can be defined as interdependence sovereignty.^10^ Societies must direct resources to organizations that facilitate shared global governance to respond to these threats nimbly and cohesively. Ironically, at a time when an organization like the WHO needs to be most supported, its major country supporter, the United States, has pulled funding for polarizing political motives.

Shared global governance must prioritize the deeply intertwined relationship between humans and natural systems, particularly when designing interventions to prepare for an ever-evolving global ecosystem. In the case of global pandemics, international organizations, such as the WHO and Global Health Security Agenda, must find ways to disseminate guidelines and spearhead initiatives to build state resilience to emerging zoonotic diseases. The G20 should lead global efforts to provide the funding required to help mitigate the global effects of COVID-19, particularly in the most vulnerable countries with weak health systems, and among the most vulnerable populations, such as migrants and refugees. Here, in the United States, a coordinated pandemic preparedness effort to align with an international response to pandemics can also be applied to other global crises requiring interdependence sovereignty, such as climate change and air pollution, which are tightly connected to societal vulnerability to pandemics. Only through shared global governance and a “planetary health” approach can we build a more resilient world prepared to mitigate the impact of this crisis and respond to the next one.
